# Exploring the untapped potential of single‐cell and spatial omics in plant biology

**DOI:** 10.1111/nph.70220

**Published:** 2025-05-21

**Authors:** Tatsuya Nobori

**Affiliations:** ^1^ The Sainsbury Laboratory University of East Anglia, Norwich Research Park Norwich NR4 7UH UK

**Keywords:** cell atlas, emerging technologies, single‐cell omics, spatial omics, temporal regulation

## Abstract

Advances in single‐cell and spatial omics technologies have revolutionised biology by revealing the diverse molecular states of individual cells and their spatial organization within tissues. The field of plant biology has widely adopted single‐cell transcriptome and chromatin accessibility profiling and spatial transcriptomics, which extend traditional cell biology and genomics analyses and provide unique opportunities to reveal molecular and cellular dynamics of tissues. Using these technologies, comprehensive cell atlases have been generated in several model plant species, providing valuable platforms for discovery and tool development. Other emerging technologies related to single‐cell and spatial omics, such as multiomics, lineage tracing, molecular recording, and high‐content genetic and chemical perturbation phenotyping, offer immense potential for deepening our understanding of plant biology yet remain underutilised due to unique technical challenges and resource availability. Overcoming plant‐specific barriers, such as cell wall complexity and limited antibody resources, alongside community‐driven efforts in developing more complete reference atlases and computational tools, will accelerate progress. The synergy between technological innovation and targeted biological questions is poised to drive significant discoveries, advancing plant science. This review highlights the current applications of single‐cell and spatial omics technologies in plant research and introduces emerging approaches with the potential to transform the field.

**Table:** 

	Contents	
	Summary	1098
I.	Introduction	1098
II.	Current application of single‐cell and spatial omics approaches in plant science	1099
III.	Emerging technologies that have potential to advance plant science	1103
IV.	Challenges and opportunities for plant research in adopting new single‐cell and spatial omics technologies	1109
V.	Conclusions and outlook	1110
	Acknowledgements	1110
	References	1110

## I. Introduction

Multicellular organisms consist of diverse, nonuniform cells, each following a distinct developmental programme and responding uniquely to environmental cues. Unraveling the molecular underpinnings of these cells and their higher order interactions that give rise to tissue functions is a critical goal in biological research. Cell biology and genomics have been the primary approaches driving these discoveries. However, traditional microscopy and bulk omics methodologies often fall short of capturing the full spectrum of cellular dynamics within tissue. For example, while fluorescence microscopy provides single‐cell and subcellular spatial resolution, it is typically limited to detecting a small number of molecular species. Similarly, bulk omics approaches comprehensively detect diverse molecules but average signals across heterogeneous cell populations, obscuring cell‐specific behaviours and spatial context. The advent of single‐cell and spatial omics technologies has transformed this landscape, unlocking the potential of cell biology and genomics, which provides unprecedented insights into cellular diversity, state transitions, and tissue architecture.

In recent years, these technologies have uncovered hidden complexities in plant biology. Single‐cell transcriptomics has mapped developmental trajectories and identified rare stress‐responsive cell populations. Spatial transcriptomics has illuminated how complex organs are functionally organised. Multimodal analyses that span different layers of molecular events are beginning to unravel the regulatory mechanisms that drive cell‐type‐specific responses. These advances highlight the power of single‐cell and spatial omics in studying plant development (Nolan & Shahan, 2023) and stress responses (Tenorio Berrío & Dubois, 2024).

**Fig. 1:**
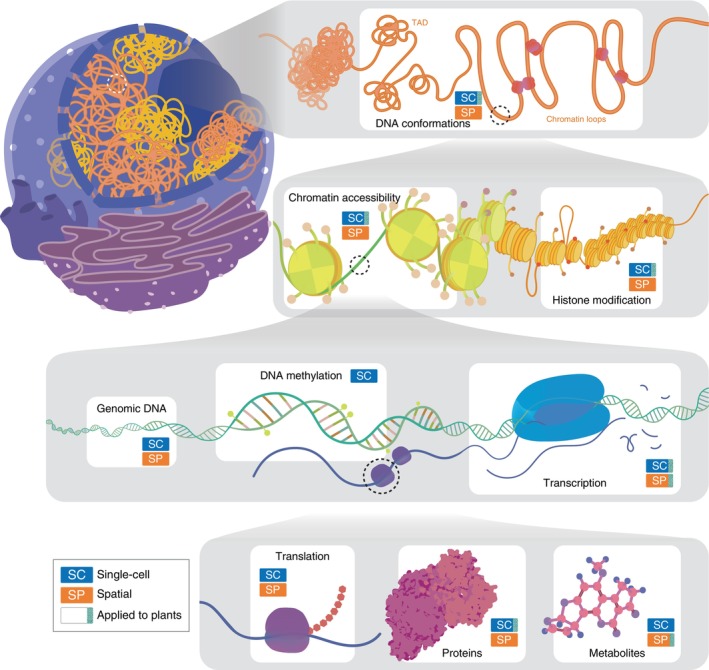
Various modalities for measurement with single‐cell and spatial omics. Single‐cell and spatial omics methods have been developed to cover various layers of molecular events in a cell. These include 3D chromatin architecture, DNA methylation, chromatin modifications, chromatin accessibility, transcript abundance, translation activity, protein abundance, and metabolite abundance. Approaches adopted in plants are indicated. TAD, topologically associated domain.

Despite these achievements, the adoption and development of single‐cell and spatial omics technologies in plant research have followed a different trajectory compared with animal and human studies, shaped by unique technical challenges, biological constraints, and research priorities. While single‐cell RNA‐sequencing and chromatin accessibility profiling have been widely adopted in plant research, many other powerful single‐cell and spatial technologies remain underutilised (Fig. 1). These untapped methods – such as simultaneous profiling of multiple modalities, comprehensive spatiotemporal profiling, and high‐content single‐cell genetic perturbations – hold great potential for advancing plant biology by providing deeper and more integrative perspectives on cellular functions.

This review explores diverse single‐cell and spatial omics technologies, focussing on emerging methods that await broader adoption in plant research. I begin by highlighting recent applications that have yielded novel biological insights and then explore promising technologies developed in other fields that could impact plant research. By presenting this technological landscape, I aim to help catalyse new applications and accelerate methodological innovation in plant science. I also address plant‐specific practical considerations while identifying opportunities for technological advancement to overcome current limitations.

## II. Current application of single‐cell and spatial omics approaches in plant science

### 1. Single‐cell omics

#### Transcriptome

Single‐cell (sc) and single‐nucleus (sn) transcriptomics is a key entry point into single‐cell omics for plant researchers. Most sc/snRNA‐seq methods can be classified into two main categories: droplet‐based and well‐based approaches. The droplet‐based method, particularly the 10× Genomics platform, is widely adopted. Well‐based methods include Smart‐seq (Ramsköld *et al*., 2012), which can analyse full‐length transcriptome with isoform resolution, and combinatorial indexing, which uses ‘split and pool’ strategies for transcript barcoding. While combinatorial indexing achieves increased cell throughput, droplet‐based methods typically provide greater molecular depth per cell/nucleus. For example, Swift *et al*. (2024) observed that a droplet‐based platform captured *c*. 50% more unique molecular identifiers per cell than a combinatorial indexing approach in rice and sorghum. An emerging alternative, particle‐templated instant partition sequencing, simplifies cell isolation using vortexing in water–oil emulsions instead of microfluidics, providing scalability and flexibility (Clark *et al*., 2023). This method may be beneficial for plant tissues, in which cell wall debris can interfere with microfluidic‐based techniques.

sc/snRNA‐seq has been instrumental in tracing developmental trajectories within cell types and uncovering previously uncharacterised intermediate states, some of which are rare and undetectable by conventional methods (Birnbaum, 2018; Nolan & Shahan, 2023). For instance, Roszak *et al*. (2021) deeply characterized the developmental trajectory of Arabidopsis protophloem, which occurs in as little as 19 cells. Beyond development, these techniques have revealed novel cell states under stress (Tenorio Berrío & Dubois, 2024). For instance, pathogen infection is spatially heterogeneous and temporally dynamic, giving rise to multiple cellular states within infected tissues. Using scRNA‐seq, Tang *et al*. (2023) discovered a specific cell type with elevated expression of a particular class of immune components, whereas Zhu *et al*. (2023) observed a single developmental cell type diverging into two distinct states, expressing resistance‐ or susceptibility‐related genes. Furthermore, sc/snRNA‐seq has facilitated cross‐species cell‐type comparisons at the molecular level, rather than focussing solely on anatomical features. Guillotin *et al*. (2023) compared root cell types across three grass species (maize, sorghum, and Setaria), demonstrating how whole‐genome duplication and the recruitment of gene modules can drive cell‐type divergence. Swift *et al*. (2024) compared a C_4_ plant (sorghum) and a C_3_ plant (rice) for their cell‐type‐specific responses to light, identifying substantial differences in gene expression within the bundle sheath and offering insights into the cellular and molecular evolution of C_4_ photosynthesis. Overall, sc/snRNA‐seq has provided unprecedented opportunities to identify and analyse cell types and states. This has also stimulated discussions on what cell types/states are, which is currently a highly debated topic and covered elsewhere (Amini *et al*., 2023; Rusnak *et al*., 2024).

#### Chromatin accessibility

Chromatin accessibility is another key modality commonly studied at the single‐cell level in plants. Single‐cell chromatin accessibility analysis is typically performed using a single‐nucleus Assay for Transposase‐Accessible Chromatin using sequencing (snATAC‐seq) (Buenrostro *et al*., 2015). Both droplet‐based and well‐based approaches have been successfully implemented in plant systems. snATAC‐seq alone has classified major cell types across multiple plant tissues and species, while integrating it with scRNA‐seq has identified previously indistinguishable subtypes (Dorrity *et al*., 2020). In Arabidopsis and maize, snATAC‐seq revealed that approximately one‐third of accessible chromatin regions (ACRs) are cell‐type‐specific (Dorrity *et al*., 2020; Marand *et al*., 2021). In maize, cell‐type‐specific ACRs are more frequently associated with phenotypic variation (Marand *et al*., 2021). Notably, scATAC‐seq has expanded the understanding of distal ACRs – regions beyond the traditionally studied 1‐ to 2‐kb upstream of transcription start sites – demonstrating their functional relevance. For instance, many distal ACRs are cell‐type‐specific (Marand *et al*., 2021), species‐specific (Yan *et al*., 2024), or enriched in key C_4_ photosynthesis enzymes (Mendieta *et al*., 2024). These findings highlight how single‐cell chromatin accessibility analyses broaden the search space for *cis*‐regulatory elements relevant to plant development and physiology.

#### Proteomics

Proteomic analyses offer direct insights into cellular functions and states, complementing transcriptomic and epigenomic data. Driven by advances in experimental design, sample preparation, data acquisition, and analysis, accurate quantification of thousands of proteins across thousands of single cells is now feasible in mammalian cells (Gatto *et al*., 2023). Two recent studies have demonstrated successful single‐cell proteomics in plants (Fulcher *et al*., 2024; Montes *et al*., 2024). Montes *et al*. (2024) employed fluorescence‐activated sorting of individual root protoplasts into wells for cell lysis, protein digestion, isotope labelling, and multiplexing, followed by LC‐MS/MS analysis. This approach identified 3217 proteins from 756 endodermis and cortex protoplasts, successfully distinguishing these cell types based on protein expression. Fulcher *et al*. (2024) employed nanoPOTS sample preparation for label‐free single‐cell proteomics of Arabidopsis leaf mesophyll cells, detecting over 3000 proteins from *c*. 100 protoplasts under control and drought stress conditions. Although single‐cell proteomics holds promise for plant research (Anderton & Uhrig, 2025), a major limitation is the requirement for protoplasting, which may not be feasible for various tissue types and could substantially alter the proteomic landscape. To avoid such problems, protoplast‐free alternative approaches are worth considering. For instance, although not at the single‐cell resolution, proximity labelling can label proteins in specific cell populations if the proximity labelling enzymes are specifically expressed in target cells (opportunities and challenges discussed in Box 1). Wallner *et al*. (2024) demonstrated this approach by expressing TurboID using promoters specific to guard cell developmental stages to achieve cell‐type‐ and state‐specific proteome analyses. Single‐nucleus proteomics is in theory possible (Derks *et al*., 2024), although low abundance of nuclei proteins remains a key challenge.

Box 1 **Is single‐cell resolution absolutely necessary?**

The strength of single‐cell omics lies in its *post hoc* flexibility to group cells into populations or align them along developmental trajectories without prior knowledge. However, the full range of single‐cell omics technologies remains out of reach for many plant biologists due to high costs and sample‐specific challenges. This raises an important question: are single‐cell analyses essential for addressing our biological questions?

In some cases, single‐cell analysis can be bypassed. For example, if research focusses on a specific cell population, there are several effective methods to enrich these cells or their molecular contents. Fluorescence‐activated cell sorting with fluorescently labelled marker lines is widely used in plant biology and is compatible with transcriptomic, proteomic, metabolomic, and epigenome analyses (Birnbaum *et al*., 2005; Petricka *et al*., 2012; Moussaieff *et al*., 2013; Kawakatsu *et al*., 2016; You *et al*., 2017). Additionally, cell‐type‐specific proteomics can be achieved through proximity labelling within targeted cell types (Wallner *et al*., 2024), while translating ribosome affinity purification sequencing enables translatome profiling by capturing ribosome‐bound transcripts from specific cell types (Mustroph *et al*., 2009).

These enrichment strategies are informative and more accessible than single‐cell omics. However, they fail to capture molecular and morphological heterogeneity within the targeted cell population, mostly transcriptionally defined. Single‐cell and spatial approaches are indispensable for uncovering this deeper layer of cellular diversity across different molecular modalities.

Critically, these enrichment methods rely on prior knowledge of cell population‐specific gene regulatory elements, such as promoters and enhancers. Building a comprehensive catalogue of regulatory elements will greatly expand our ability to accurately access a broader range of cell populations. This requires unbiased single‐cell transcriptomic and epigenomic analyses paired with detailed spatial validation across diverse tissue types. Therefore, community‐driven efforts to develop reference cell atlases are vital for advancing our understanding of cellular heterogeneity in plant tissues.

#### Multiomics

Single‐cell multiomics simultaneously measures multiple molecular features within individual cells, offering advantages over integrating data from separate single‐modality experiments, which pose computational challenges (Argelaguet *et al*., 2021). Additionally, single‐cell multiome data can serve as a ground truth to which single‐modality data can be aligned. The combination of scRNA‐seq and scATAC‐seq is the most widely used multimodal approach, available in both droplet‐based and well‐based platforms (Vandereyken *et al*., 2023). This approach directly correlates ACR changes with gene expression at the single‐cell level, offering insights into gene regulatory mechanisms. The plant biology field has successfully adopted 10× Genomics single‐nucleus multiomics (snMultiomics) for RNA + ATAC analysis. Liu *et al*. (2024) applied snMultiomics to Arabidopsis seedlings under drought stress, revealing potential cell‐type‐specific gene regulatory networks responsive to the stress. Nobori *et al*. (2025) analysed Arabidopsis leaves infected by bacterial pathogens, uncovering transcription factor (TF)‐ACR‐gene modules activated in specific cell populations during immunity. Notably, there are cases in which changes in chromatin accessibility do not correlate with gene expression; roughly half of immune‐responsive genes showed a lack of correlation with nearby ACRs (Nobori *et al*., 2025), suggesting the presence of additional gene regulatory mechanisms, such as histone modifications and DNA methylation of TF binding sites, highlighting the need to profile other modalities.

### 2. Spatial omics

#### Spatial transcriptomics

**Fig. 2:**
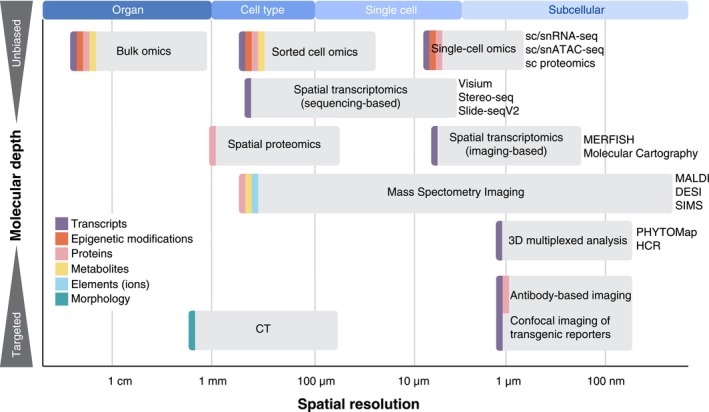
Molecular coverage vs spatial scale in various methods applied to plant research. The coloured ribbons indicate the types of molecular information each method analyses. This figure was inspired by Jain *et al*. (2023). CT, computed tomography; DESI, desorption electrospray ionization; MALDI, matrix‐assisted laser desorption/ionisation; SIMS, secondary ion mass spectrometry.

While single‐cell omics provide unprecedented insights into cellular heterogeneity, they lack spatial context. Traditionally, cell population‐specific gene expression has been validated via transgenic reporter lines, but this approach is time‐consuming and unable to simultaneously visualize many genes, constraining our ability to annotate the growing list of cell populations and understand their spatial relationships. Spatial transcriptomics has emerged as a powerful solution to this limitation. There are two main approaches to spatial transcriptomics: sequencing‐based and imaging‐based (Moses & Pachter, 2022). Sequencing‐based methods use spatially barcoded arrays to capture RNA molecules from tissue sections, followed by next‐generation sequencing, with notable examples including 10× Genomics Visium, Slide‐seq (Rodriques *et al*., 2019), and Stereo‐seq (Chen *et al*., 2022). Imaging‐based methods directly visualize RNA molecules in tissue sections using fluorescently labelled oligonucleotides, exemplified by MERFISH (Chen *et al*., 2015) and seqFISH+ (Eng *et al*., 2019). While sequencing‐based methods typically offer transcriptome‐wide coverage, imaging‐based methods provide higher spatial resolution and tend to cover larger tissue areas, though the boundary between them is increasingly fading (Fig. 2).

Since its first application in plant tissues (Giacomello *et al*., 2017), spatial transcriptomics has demonstrated success in plant research (Cox *et al*., 2021). Sequencing‐based methods including Visium and Stereo‐seq have enabled cell typing in Arabidopsis leaves (Xia *et al*., 2022), tomato callus (Song *et al*., 2023), soybean seeds and nodules (Z. Liu *et al*., 2023; Zhang *et al*., 2024a), poplar roots (Lv *et al*., 2024), barley grain (Peirats‐Llobet *et al*., 2023), maize ears and kernels (Fu *et al*., 2023; Wang *et al*., 2024), and simultaneous profiling of Medicago root transcripts alongside colonising beneficial microbes (Serrano *et al*., 2024). Imaging‐based methods – *in situ* sequencing, molecular cartography, and MERFISH – have been successfully applied to different plant tissues, including maize meristems (Laureyns *et al*., 2022; Perico *et al*., 2024; Xu *et al*., 2024), maize roots (Guillotin *et al*., 2023), Arabidopsis leaves (Nobori *et al*., 2025), Arabidopsis siliques (Lee *et al*., 2023), wheat inflorescence (Long *et al*., 2024; Xu *et al*., 2025), and soybean nodules (Cervantes‐Pérez *et al*., 2024).

Integrating single‐cell omics with spatial transcriptomics offers exciting opportunities to capture complex cellular responses within tissue contexts. Z. Liu *et al*. (2023) combined snRNA‐seq with Stereo‐seq in soybean nodules to identify transitional cell states within uninfected cells. Serrano *et al*. (2024) integrated snRNA‐seq with Visium in mycorrhiza‐colonised Medicago to uncover cell‐type‐ and infection‐specific states. Nobori *et al*. (2025) combined snMultiomics and MERFISH in pathogen‐infected Arabidopsis leaves to reconstruct spatiotemporal dynamics of immune responses, revealing rare immune cell states and their potential regulatory mechanisms.

### 3. Single‐cell and spatial atlases

Large efforts have been made to create molecular and spatial cell atlases of various organisms (Quake, 2022). These unified atlases serve as invaluable community resources, guiding focussed analyses of specific cell populations and facilitating the development of new atlases across modalities, treatments, and genotypes, supported by advanced computational reference mapping methods (Lotfollahi *et al*., 2024). The Human Cell Atlas exemplifies the power of this approach, having proved instrumental in bridging gaps between genotypes and disease phenotypes by identifying specific cell types, states, programmes, and contexts (Rood *et al*., 2022). Inspired by these successful approaches, similar comprehensive atlases have recently become available in model plants, including the transcriptome atlases of Arabidopsis (Lee *et al*., 2023; X. Guo *et al*., 2025) and soybean (Cervantes‐Perez *et al*., 2024; Fan *et al*., 2025), the chromatin accessibility atlases of maize and rice (Marand *et al*., 2021; Yan *et al*., 2024), and transcriptome and chromatin accessibility atlases of soybean (Cervantes‐Perez *et al*., 2024; Zhang *et al*., 2024a). These observational atlases help with the molecular definition of cell types and states across diverse organs and plant species, enabling deeper investigations into plant biology at the cellular scale (Box 1).

Over the past decade, single‐cell and spatial omics technologies have revolutionised plant biology by enabling molecular analyses at the resolution of individual cells and cell populations. These approaches overcome previous limitations that confined studies to specific cell types or a limited set of molecules, providing a comprehensive view of cellular diversity and function. As these technologies continue to evolve, the field is shifting from primarily exploratory and descriptive studies towards more hypothesis‐driven and application‐focussed research, supported by improved protocols and increasingly comprehensive reference atlases. Additionally, the field is poised to adopt emerging technologies proven successful in nonplant systems, which will be discussed in the following section.

## III. Emerging technologies that have potential to advance plant science

While sc/snRNA‐seq, snATAC‐seq, and spatial transcriptomics have gained popularity in plant biology, numerous other modalities and techniques remain underexplored. This section highlights emerging and promising approaches, focussing on their technical aspects rather than on their specific biological applications.

### 1. Sample multiplexing

Profiling larger numbers of cells enhances statistical power for identifying rare populations and enables simultaneous analysis of multiple conditions. Various multiplexing techniques exist for droplet‐based sc/snRNA‐seq, including cell hashing with DNA‐labelled antibodies (McGinnis *et al*., 2019) or lipid‐tagged indices (Guo *et al*., 2019), and pooling genetically distinct samples for natural variation‐based demultiplexing (Kang *et al*., 2017). Single‐cell combinatorial fluidic indexing (scifi) RNA‐seq integrates whole transcriptome pre‐indexing with droplet‐based single‐cell RNA‐seq (scRNA‐seq), achieving an order of magnitude higher throughput while maintaining transcriptome complexity (Datlinger *et al*., 2021). Similarly, UDA‐seq first labels cells using droplet microfluidics followed by well‐based indexing (Li *et al*., 2025). Inspired by scifi‐RNA‐seq, Zhang *et al*. (2024b) developed scifi‐ATAC‐seq, which enables profiling of up to 200 000 nuclei from multiple plant species in a single run through pre‐indexing DNA with barcoded Tn5 transposase. Marand *et al*. (2024) pooled multiple maize genotypes followed by *in silico* genotyping to assay *c*. 1.37 million nuclei across 172 distinct maize inbred lines, revealing cell‐type‐specific rewiring of gene regulatory networks driven by adaptation. Highly multiplexed sc/snRNA‐seq and snATAC‐seq will enable the testing of various treatments and genetic perturbations to better understand cell‐type/state diversity and gene regulatory landscapes.

### 2. Various RNA species

Single‐cell and spatial transcriptome methods applied to plants predominantly assay protein‐coding polyadenylated RNAs, missing various types of noncoding RNAs that play crucial regulatory roles (Yu *et al*., 2019; Chorostecki *et al*., 2023). Additionally, most coding RNA assays focus on the either 5′ or 3′ ends, lacking the full‐length transcript information necessary for understanding post‐transcriptional modifications such as alternative splicing – an important mechanism in plant development and stress responses (Tognacca *et al*., 2023).

Recently, methods have emerged to analyse diverse RNA species, including noncoding and full‐length transcripts, at single‐cell or spatial resolution, primarily in animal systems (Maji *et al*., 2025). Smart‐seq3, a plate‐based method, captures full‐length transcripts with high sensitivity, enabling allele and isoform analysis (Hagemann‐Jensen *et al*., 2019). MAS‐ISO‐seq employs long‐read sequencing for full‐length single‐cell transcriptome profiling (Al'Khafaji *et al*., 2023). Smart‐seq‐total extends Smart‐seq by adding polyA tails to noncoding RNAs, facilitating total RNA capture from single cells (Isakova *et al*., 2021). Vast transcriptome analysis of single cells by dA tailing (VASA‐seq) adapts Smart‐seq‐total to a droplet‐based format, increasing cell throughput (Salmen *et al*., 2022). Spatial total RNA sequencing integrates *in situ* polyadenylation with Visium spatial transcriptomics to analyse both coding and noncoding RNAs, including viral RNAs, *in situ* (McKellar *et al*., 2022).

In plants, Brosnan *et al*. (2019) expressed AGO1 in specific cell types to enrich miRNAs loaded onto AGO1, enabling cell‐type‐specific miRNAome analysis in Arabidopsis root tips. This study revealed widespread miRNA cell‐type specificity, suggesting a role for miRNA‐mediated silencing in fine‐tuning gene expression in a cell‐type‐specific manner. Long *et al*. (2021) introduced FlsnRNA‐seq, a full‐length single‐nucleus RNA profiling method for plants, facilitating the analysis of alternative splicing and polyadenylation in a cell‐type‐specific manner.

### 3. Translational regulation

Translation is a key process in gene expression and is essential for regulating plant development and responses to the environment. While genome‐wide translation profiling in bulk plant tissues has revealed diverse regulatory mechanisms, including those involving upstream open reading frames (H‐Y. L. Wu *et al*., 2024), comprehensive cell‐type‐ and state‐specific translational regulation in plants remains largely unexplored. Plants have expanded translation initiation complexes and ribosomal proteins, which were proposed to provide heterogeneity in translational machinery (H‐Y. L. Wu *et al*., 2024). Understanding the cellular basis of such potential heterogeneity is an important open question.

Recently, two single‐cell translatome methods have been developed and applied in mouse tissues. Single‐cell Ribo‐seq uses micrococcal nuclease to digest ribosome‐unprotected RNAs, generating ribosome‐protected footprints for sequencing (Van Insberghe *et al*., 2021). Similarly, Ribo‐ITP employs RNase I to enrich ribosome‐protected RNAs through microfluidic isolation, enabling RNA recovery from ultra‐low‐input samples (Ozadam *et al*., 2023). In the spatial domain, RIBOMap visualizes thousands of ribosome‐occupied RNAs *in situ*, revealing cell‐type‐ and region‐specific translational regulation in the mouse brain (Zeng *et al*., 2023).

Translating ribosome affinity purification followed by sequencing (TRAP‐seq) offers an alternative to single‐cell resolution methods. Translating ribosome affinity purification followed by sequencing involves expressing epitope‐tagged ribosomal proteins to affinity‐purify assembled ribosomes and their associated mRNAs for sequencing (Mustroph *et al*., 2009). When combined with cell‐type‐ or state‐specific gene regulatory elements, TRAP‐seq enables targeted translatome analysis of specific plant cell populations (Kajala *et al*., 2021) (Box 1). Identifying novel regulatory elements through single‐cell transcriptome and epigenome analyses promises to expand these applications.

### 4. Epigenome analyses

#### DNA methylation

DNA methylation serves as a critical epigenetic mechanism that influences gene expression, cellular differentiation, and genomic stability in both plants and animals (Luo *et al*., 2018; Gallego‐Bartolomé, 2020). For single‐cell analyses, the most widely used approach is bisulfite sequencing approaches (Tian *et al*., 2023; Liu *et al*., 2023c), in which unmethylated cytosines are chemically converted to uracils while methylated cytosines remain unchanged, allowing base‐resolution and genome‐wide analyses. A bisulfite conversion‐based single‐cell methylome assay has been commercialised by ScaleBio. Enzymatic methyl sequencing approaches have been developed as less destructive alternatives (Nichols *et al*., 2024). Atlas‐scale single‐cell methylome datasets generated in mouse and human brains have demonstrated the power of single‐cell methylome profiling in identifying cell types and regulatory elements (Liu *et al*., 2023c; Tian *et al*., 2023). However, achieving comprehensive methylome coverage in single cells while managing costs and technical complexity (low‐volume liquid handling for library preparation) remains challenging.

Although single‐cell DNA methylome profiling has not been reported in plants, studies have shown that plants exhibit cell‐type‐specific DNA methylation patterns (Kawakatsu *et al*., 2016). It is plausible that cell‐specific DNA methylation changes occur during plant responses to environmental stimuli, but this remains to be explored. The discovery of unique 4‐methylcytosine specifically in Marchantia sperm cells (Walker *et al*., 2025) further emphasizes the importance of single‐cell resolution analysis. Understanding cell‐specific DNA methylation dynamics could enable the use of emerging epigenome editing tools, such as dCas9 fused with methyltransferases or demethylases (Gallego‐Bartolomé, 2020), to experimentally test causal epigenetic changes in specific cells underlying phenotypes.

#### 3D chromatin organization

3D chromatin organization provides an important regulatory layer for gene expression, but the mechanisms differ between plants and mammals. Mammalian genome organization depends heavily on CCTC‐binding factor (CTCF) and cohesin proteins to establish topologically associating domain (TAD) boundaries and chromatin loops through loop extrusion. By contrast, plants lack CTCF orthologs yet still form TAD‐like domains and chromatin loops (Ouyang *et al*., 2020). Understanding how plants achieve and regulate genome organization without these canonical factors represents a fundamental biological question, and cell‐level understanding is considered crucial to gaining further mechanistic insights (Peng *et al*., 2019).

High‐resolution single‐cell Hi‐C methods in animals have revealed significant cell‐to‐cell variation in chromatin architecture and identified rare cell populations (Jerkovic & Cavalli, 2021). Studies using single‐cell Hi‐C showed dramatic chromatin reorganization during cell differentiation and early embryonic development. For example, oocytes exhibit features of the genomic organzsation including A/B compartments, TADs, and loops when averaged over the genome, while sperm maintain a highly condensed and organised chromatin structure (Flyamer *et al*., 2017). Complementing sequencing‐based approaches, imaging methods, including oligopaint FISH with super‐resolution fluorescence microscopy (Bintu *et al*., 2018), and CRISPR‐based live imaging such as CARGO (Gu *et al*., 2018) enables direct visualization of chromatin dynamics in individual cells.

In plants, bulk Hi‐C revealed tissue‐specific differences, but averaging across cell populations masked important cell‐type variation (Ouyang *et al*., 2020). INT‐Hi‐C combines isolation of nuclei tagged in specific cell type (INTACT)‐based cell‐type enrichment with Hi‐C, identifying distinct chromatin conformations in endosperm and leaf tissues (Yadav *et al*., 2021). The first successful single‐cell Hi‐C application in plants analysed individual rice gametes, zygotes, and mesophyll cells, revealing cell‐type‐specific architectures including a ‘compact silent centre’ structure unique to eggs and zygotes that may regulate zygotic genome activation (Zhou *et al*., 2019). This study demonstrated the power of single‐cell approaches to uncover previously unknown chromatin features masked in bulk analyses in plants.

#### Chromatin modification

Histone modifications provide another crucial layer of gene regulation by affecting how tightly DNA is packed around histone octamers and the recruitment of regulatory components. For instance, environmental stresses induce active transcriptional marks, such as H3K4me2/3 or H3K9ac, and/or reduce repressive marks, such as H3K27me3, which primes stress responses in plants (Harris *et al*., 2023). Recent advances enabling single‐cell profiling of histone modifications include Cleavage Under Targets and Tagmentation (CUT&Tag) (Kaya‐Okur *et al*., 2019) and chromatin integration labelling (Harada *et al*., 2019). These approaches offer several advantages over traditional chromatin immunoprecipitation sequencing (ChIP‐seq), including lower input requirements and improved signal‐to‐noise ratios, making them suitable for single‐cell applications. In animal systems, single‐cell CUT&Tag has revealed cell‐type‐specific histone modification patterns and their relationships with gene expression states (Bartošovič *et al*., 2021). The study also analysed scCUT&Tag of TFs, which showed sparser data than histone modifications due to lower abundance but revealed cell‐type‐specific TF bindings. More recent methods allow simultaneous profiling of multiple histone modifications (Meers *et al*., 2022; Xiong *et al*., 2024).

In plants, cell‐type‐specific analysis of histone modifications was performed by isolating shoot apical meristems (SAMs) using INTACT followed by ChIP‐seq, identifying SAM‐specific chromatin states during flowering (You *et al*., 2017). More recently, the application of CUT&Tag showed some promise in low‐input or single‐cell analyses of histone modifications in plant tissues (Tao *et al*., 2020; Ouyang *et al*., 2022). Like DNA methylation editing, histone modifications can also be manipulated by using dCas9 or SunTag systems in plants (Oberkofler & Bäurle, 2022; Wang *et al*., 2023), opening the door for exploring causal histone modifications in specific cell populations.

### 5. Single‐cell multiomics

**Fig. 3:**
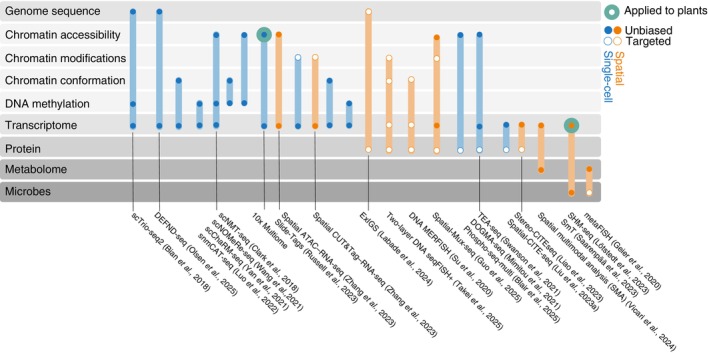
Combinations of modalities that can be simultaneously profiled in existing single‐cell or spatial omics methods. Methods applied to plant tissues are indicated. References are shown for selected technologies.

While single‐cell RNA and ATAC‐seq coprofiling have been successfully applied in plant biology, the single‐cell multiomics methods have expanded to include various other combinations, primarily in animal systems (Fig. 3). Protein‐centric approaches have made significant strides through antibody‐based methods such as cellular indexing of transcriptomes and epitopes (CITE‐seq) (Stoeckius *et al*., 2017) for cell surface proteins, and newer techniques, Single‐Cell Protein And RNA Co‐profiling (Reimegård *et al*., 2021) and inCITE‐seq (Chung *et al*., 2021), for intracellular and intranuclear epitopes, respectively. Phospho‐seq profiles phosphorylated cytoplasmic and nuclear proteins in conjunction with chromatin accessibility (Blair *et al*., 2025). Higher modality approaches, Transcription, Epitopes, and Accessibility (TEA)‐seq, DOGMA‐seq, and Phospho‐seq‐multi, now enable simultaneous profiling of antibody‐labelled proteins, RNA, and chromatin accessibility (Mimitou *et al*., 2021; Swanson *et al*., 2021; Blair *et al*., 2025). To date, these multiomic approaches have not been applied in plants, in large part due to technical limitations such as the limited availability of antibodies and challenges in sample preparation (Section IV).

Multimodal methods focussing on DNA and RNA have also evolved rapidly. These include simultaneous profiling of chromatin architecture and transcriptome (LiMCA, H. Wu *et al*., 2024) or DNA methylation (scMethyl‐HiC, Li *et al*., 2019; snmC‐seq, Lee *et al*., 2019), genome sequence and transcriptome (DNA and expression following nucleosome depletion (DEFND)‐seq, Olsen *et al*., 2025), transcriptome and chromatin modifications (paired‐Tag, Xie *et al*., 2023), as well as triple‐omics approaches including scNMT‐seq (Clark *et al*., 2018), snmCAT‐seq (Luo *et al*., 2022), scNOMeRe‐seq (Wang *et al*., 2021b), scChaRM‐seq (Yan *et al*., 2021), and scTrio‐seq2 (Bian *et al*., 2018) that combine RNA, methylation, and chromatin accessibility measurements or genome sequence. For a more comprehensive list of technologies, I refer to recent review articles (Baysoy *et al*., 2023; Vandereyken *et al*., 2023). These multimodal approaches not only refine cell‐type classification but also provide crucial insights into cell population‐specific gene regulatory mechanisms by revealing relationships between different molecular layers (Badia‐I‐Mompel *et al*., 2023), an area largely unexplored in plants.

### 6. Advanced spatial omics

#### 3D spatial analysis of thick tissues

Current spatial transcriptomics typically analyses thin tissue sections, but a full understanding of cellular diversity and spatial relationships requires 3D resolution. Two main approaches have emerged: serial sectioning with 3D reconstruction and direct thick tissue imaging.

Serial sectioning achieves 3D reconstruction through analysing multiple thin sections covering thick tissue volumes. Sampath Kumar *et al*. (2023) applied Slide‐seq to a whole mouse embryo, spanning 15–17 sections each 10 μm in thickness. Similarly, Open‐ST, which uses custom‐made arrays derived from Illumina flow cells, achieved 3D reconstruction of mouse brain tissue spanning 19 sections of 10 μm in thickness, covering a total of 350 μm in depth (Schott *et al*., 2024).

Thick‐tissue imaging enables direct 3D analysis without sectioning. EASI‐FISH and cycleHCR achieved multiplexed hybridisation chain reaction (HCR) (Choi *et al*., 2018) on 300‐μm‐thick tissue (Wang *et al*., 2021a; Gandin *et al*., 2025), and Deep STARmap obtained data from 200‐μm‐thick tissue with *in situ* sequencing (Sui *et al*., 2024), profiling 24 and 1017 genes, respectively. 3D MERFISH implemented confocal imaging instead of epifluorescence imaging combined with deep learning‐based image analysis to analyse > 200 genes in up to 200‐μm‐thick samples (Fang *et al*., 2024). Challenges in imaging‐based 3D spatial transcriptomics include lower gene throughput and large data volume, requiring large computational resources.

In plants, 3D spatial transcriptomics is still emerging. HRC has been used to visualise several genes (up to 3) in whole‐mount plant tissues (Yu *et al*., 2024). PHYTOMap successfully captured cell‐type and region‐specific expression of dozens of genes (up to 28) in whole‐mount Arabidopsis root tips (Nobori *et al*., 2023), demonstrating its potential to advance 3D spatial analysis in plant biology.

#### Spatial proteomics and metabolomics

Spatial protein expression is crucial for understanding the functional organization of tissues. For example, RNAs and proteins move between cells and function or are degraded in specific cells; such information cannot be captured by mapping transcriptional activity or mRNA abundance alone. While spatial analysis of single or several proteins is well‐established, simultaneous mapping of a large number of proteins offers unique opportunities for understanding cellular functional complexity.

Spatial proteomics includes various approaches: (1) a series of immunohistochemistry‐based methods, such as cyclic immunofluorescence (cycIF) (Lin *et al*., 2018) and codetection by indexing (CODEX) (Goltsev *et al*., 2018); (2) mass spectrometry imaging (MSI); and (3) deep visual proteomics (DVP) (Mund *et al*., 2022). CycIF and CODEX use antibodies to detect multiple proteins in tissue samples, with CycIF relying on fluorescently labelled antibodies applied in cycles and CODEX utilising DNA‐barcoded antibodies with sequential fluorescent probe visualisation. MSI technologies such as matrix‐assisted laser desorption/ionisation (MALDI) integrate spatially resolved molecular ablation with MS‐based analysis of desorbed material and can capture smaller molecules, including metabolites, lipids, or peptides, although they do not achieve a comprehensive proteome analysis. DVP is the latest method that combines AI‐driven tissue image analysis and laser dissection of single cells from a complex tissue for ultrasensitive MS analysis, detecting up to 10 000 proteins with spatial resolution (Nordmann *et al*., 2024). While these approaches focus mainly on cellular‐level protein abundance, another emerging area is subcellular proteomics, which employs high‐resolution imaging (such as super‐resolution imaging) combined with multiplexed protein labelling (Schueder *et al*., 2024; Unterauer *et al*., 2024). Thul *et al*. (2017) constructed a high‐resolution subcellular map of the human proteome by antibody‐based immunofluorescence microscopy, showing that more than half of the 12 003 proteins analysed in the study were found in multiple subcellular compartments, suggesting multifunctionality of many proteins. Applying such spatial proteomics technologies in plants and developing high‐resolution proteome maps in plant cells will provide powerful resources for future discoveries in plant science.

MSI technologies enable simultaneous spatial analysis of proteins and metabolites. Besides MALDI‐MSI, desorption electrospray ionization (DESI)‐MSI and infrared laser matrix‐assisted laser DESI (IR‐MALDESI) are used for spatial metabolomics profiling. While the running cost of spatial metabolomics tends to be cheaper than other spatial omics, there remain technical challenges in the ability to detect diverse metabolites and confidently annotate the resulting data, requiring further method development (Alexandrov, 2023). Recently, an advanced cryo nanoscale secondary ion mass spectrometry simultaneously visualized multiple elements (macro‐ and micronutrients) in Arabidopsis roots at subcellular resolution, revealing a novel nutrient allocation mechanism in plants (Ramakrishna *et al*., 2025). Ge *et al*. (2025) combined spatial metabolomics with spatial transcriptomics and scRNA‐seq to reveal molecular dynamics in cotton somatic embryos. MSI applications in plant biology show promise while presenting tissue‐specific challenges (Yin *et al*., 2024).

#### Spatial multiomics

Spatial omics is increasingly multimodal, integrating transcriptomics with other molecular layers for deeper insights (Fig. 3). RNA‐MERFISH was combined with DNA‐MERFISH to simultaneously profile over 1000 genes and more than 1000 genomic loci (Su *et al*., 2020). DNA seqFISH+ was extended to allow simultaneous profiling of over 3000 genomic loci along with 17 chromatin marks and subnuclear structures by sequential immunofluorescence and the expression profile of 70 RNAs (Takei *et al*., 2021). This approach was further advanced using an updated barcoding strategy to simultaneously profile over 100 000 genomic loci along with more than 10 000 genes and dozens of immunofluorescence labels (Takei *et al*., 2025). When applied to plants, these methods will provide new insights such as cell‐type‐specific organization of chromatin compartments in the nucleus and its impact on gene regulation.

Other technologies, such as Slide‐Tags, have enabled spatial barcoding of nuclei in tissue sections followed by single‐nucleus multiome assays for RNA‐seq and ATAC‐seq (Russell *et al*., 2023). DBiT‐seq, a sequencing‐based spatial omics method (Liu *et al*., 2020), demonstrated high adaptability to multimodal analysis, such as in spatial CUT&Tag–RNA‐seq to study histone modifications together with the transcriptome, as well as spatial ATAC–RNA‐seq to reveal concordant and discordant patterns between the spatial epigenome and transcriptome in mouse and human brains (Zhang *et al*., 2023). Spatial‐CITE‐seq was used to coprofile the whole transcriptome along with 189 and 273 proteins in mouse and human tissues, respectively (Y. Liu *et al*., 2023). Stereo‐seq was also combined with CITE‐seq (Stereo‐CITE‐seq) to profile untargeted spatial transcriptomes along with more than 10 immunofluorescence staining (Liao *et al*., 2023). Spatial‐Mux‐seq successfully profiled the whole transcriptome, chromatin accessibility, two histone modifications, and a panel of 122 proteins in a mouse brain (P. Guo *et al*., 2025).

Beyond the integration of transcriptomics with DNA and/or protein analysis, spatial multiomics also includes the combination of metabolomics with transcriptomics. For example, Vicari *et al*. (2024) combined Visium with MALDI‐MSI to analyse both spatial transcriptomics and metabolomics. Complementary to highly multiplexed metabolite analyses, biosensors for various small molecules, such as phytohormones, are available for plants, with ongoing advancements in biosensor engineering (Castaneda‐Méndez *et al*., 2024). These biosensors enable high‐resolution, 3D, and real‐time imaging and have the potential to be integrated with other imaging modalities, including RNA and protein visualization.

The use of multimodal spatial omics approaches is also expanding to include host‐associated microbes (Lötstedt *et al*., 2023; Saarenpää *et al*., 2023), linking host gene expression with the spatial distribution of microbiomes. Geier *et al*. (2020) developed metaFISH, which combines MALDI‐MSI with *in situ* hybridisation targeting bacterial 16S rRNA, spatially linking metabolic phenotypes with microbiome distribution in host tissues. While these methods do not provide single‐cell resolution for microbes, there are emerging approaches, such as HyPR FISH (Shi *et al*., 2020), for profiling bacterial distribution, and microbial seqFISH (Dar *et al*., 2021) or microbial MERFISH (Sarfatis *et al*., 2024), for spatially mapping microbial gene expression at the single‐cell level. When combined with imaging‐based spatial omics of the host, these methods have the potential to resolve individual cells within a holobiont.

#### Expansion microscopy

Expansion microscopy (ExM), a sample preparation technique that physically enlarges biological specimens by embedding them in a swellable hydrogel, is a powerful solution to overcome the diffraction limit that constrains traditional imaging‐based spatial transcriptomics, especially in tissues with high transcript density. This technique has been successfully integrated with various spatial transcriptomics approaches to enhance both multiplexing capacity and subcellular resolution in mammalian tissues. MERFISH and seqFISH+ with ExM (Eng *et al*., 2019; Xia *et al*., 2019) profiled *c*. 10 000 genes. ExSeq is an untargeted method, which has demonstrated the detection of over 3000 genes (Alon *et al*., 2020). Combined with array‐based spatial transcriptomics, Ex‐ST achieved higher spatial resolution while retaining the untargeted capturing of RNAs (Fan *et al*., 2023). These methods have revealed important insights into subcellular RNA compartmentalisation. Most recently, ExM has been adapted for *in situ* genome sequencing through the development of ExIGS (Labade *et al*., 2024), which enables simultaneous visualisation of nuclear proteins at nanoscale resolution in 3D, providing unprecedented insights into genome positioning and function within the nucleus. ExM has been successfully applied to plant tissues (Kao & Nodine, 2019; Hawkins *et al*., 2023; Cox Jr *et al*., 2024; Gallei *et al*., 2025; Grison *et al*., 2025), opening new avenues for enhanced spatial omics experiments in plant biology.

### 7. Temporal information and lineage tracing

While most single‐cell and spatial omics technologies provide static snapshots, understanding cellular lineages and molecular histories is crucial for comprehending dynamic biological processes. Temporal information can be retrieved through endogenous molecular information and engineered recording systems.

Endogenous approaches assume that single‐cell omics capture cells at different stages of dynamic processes, enabling computational reconstruction of cellular trajectories. This principle underlies pseudotime analyses and RNA velocity (Saelens *et al*., 2019; Bergen *et al*., 2021), now standard in plant research. Waddington–Optimal Transport reconstructs cellular trajectories from time course scRNA‐seq data (Schiebinger *et al*., 2019), which was successfully applied to study root development (Nolan *et al*., 2023). Another approach uses natural somatic mutations, including single‐nucleotide variants, copy number variations, transposon insertions, and mtDNA mutations, as endogenous DNA barcodes for understanding cell division histories (McConnell *et al*., 2013; Ludwig *et al*., 2019). In plant research, natural somatic mutations have revealed early separation of leaf and root cell lineages in shrub willow (Ren *et al*., 2021).

**Fig. 4:**
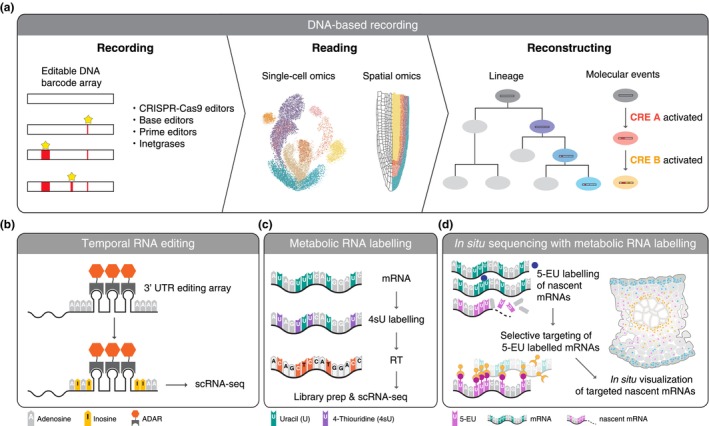
Temporal resolution in single‐cell and spatial omics. (a) DNA‐based methods to record cell lineage and the history of responses. (b) RNA timestamps record temporal dynamics of transcription by recruiting adenosine deaminase acting on RNA (ADAR) to target RNAs, which induces A‐to‐I editing. (c, d) Metabolic labelling combined with single‐cell RNA‐seq (c) *in situ* sequencing (d). CRE, *cis*‐regulatory element; 4sU, 4‐thiouridine; 5‐EU, 5‐ethynyluridine.

Engineering approaches expand the amount and resolution of cellular history that can be recorded and read by single‐cell and spatial omics technologies. Various new technologies have been developed to record cellular history into DNAs, RNAs, and proteins, with DNA‐based recording systems being particularly actively developed (Askary *et al*., 2024) (Fig. 4a). The most common way of DNA recording is CRISPR‐Cas9 editing of ‘storage’ DNA regions, which enables high‐resolution lineage tracing at organ and organism levels (McKenna *et al*., 2016; Spanjaard *et al*., 2018). These approaches can be combined with scRNA‐seq to simultaneously reveal cell‐type information alongside lineage relationships (Alemany *et al*., 2018; Raj *et al*., 2018). DNA Typewriter implemented a prime editor to enable continuous recording of molecular events while storing the order of events and reconstructing cell lineage over weeks (Choi *et al*., 2022). Lineage tracing has further expanded to include spatial contexts. MEMOIR (Frieda *et al*., 2017) and Spatial iTracer (He *et al*., 2022) employ CRISPR‐based barcoding, which is spatially read out with smFISH and sequencing‐based spatial transcriptomics, respectively. intMEMOIR employs integrase‐based lineage recording to expand the number of states it can record, which is decoded via five sequential rounds of HCR (Chow *et al*., 2021). More recently, baseMEMOIR employs base editing instead of CRISPR‐Cas9 or integrases, further expanding the number of recordable states (Chadly *et al*., 2024). Advancing these methods could allow researchers to record every cell division within a single individual in the near future.

In plants, CRISPR‐based lineage tracing strategies were recently implemented. Donà *et al*. (2023) developed a system to fluorescently label specific cells by inducing CRISPR gene editing and performed lineage tracing of Arabidopsis shoot and *Marchantia polymorpha* apical notch. Lu *et al*. (2024) developed an inducible DNA barcoding system, combining CRISPR barcoding and multifluorescence genetic cell‐labelling approaches to enable high‐resolution lineage tracing in Arabidopsis. This study showed that plant regeneration originates from a single somatic cell within a donor tissue, whereas lateral root development arises from multiple cells (Lu *et al*., 2024).

Recording past molecular events (such as gene expression) is another aspect of temporal understanding. DNA Typewriter can be combined with ENGRAM, which records the history of the activities of *cis*‐regulatory elements to a DNA tape in mammalian cells (Chen *et al*., 2024). This method can potentially be combined with single‐cell and spatial omics methods to capture the molecular history of cells in space. In plants, integrase‐based molecular recording has been applied to record gene expression events at the single‐cell level *in situ* (Guiziou *et al*., 2023; Maranas *et al*., 2024).

RNA timestamps employ adenosine deaminase acting on RNA (ADAR) to introduce A‐to‐I edits that accumulate over time, which are then measured with scRNA‐seq, thereby reconstructing the history of transcription events (Rodriques *et al*., 2021) (Fig. 4b). Integrating scRNA‐seq with metabolic RNA labelling enables time‐resolved monitoring of transcriptional responses in various cell types (Erhard *et al*., 2022) (Fig. 4c). TEMPOmap combines 5‐ethynyl uridine (EU) labelling with *in situ* sequencing for multiplexed detection of labelled RNAs in space (Ren *et al*., 2023) (Fig. 4d). Metabolic labelling of bulk plant tissues has revealed differing RNA half lives in Arabidopsis (Szabo *et al*., 2020). Whether such differences occur across or within specific cell types remains an open question, addressable with single‐cell methods.

Together, applying advanced lineage tracing and event recording methods in plants will reveal novel cell differentiation events with underlying regulatory mechanisms and enable precise biophysical modelling of dynamic multicellular systems.

### 8. Genetic and chemical perturbation

**Fig. 5:**
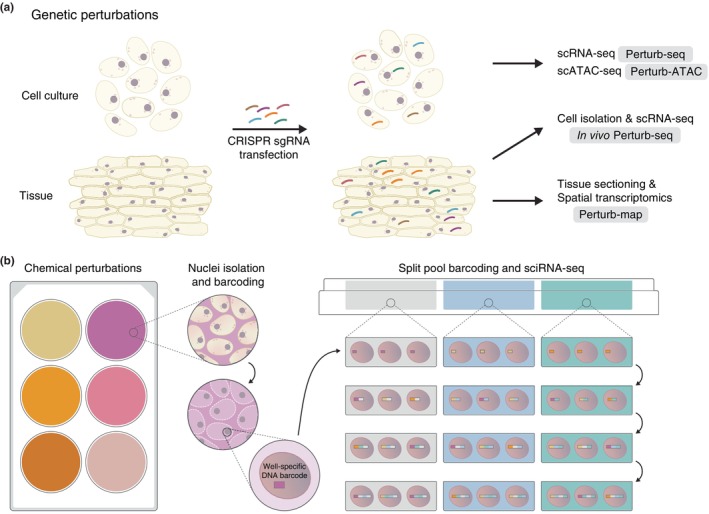
Genetic and chemical perturbations. (a) High‐content single‐cell and spatial genetic perturbation assays. (b) Single‐cell transcriptome screening of cells treated with various chemicals. Nuclei isolated from chemical‐treated cells are uniquely barcoded. Single‐cell RNA‐seq libraries are generated by sequential barcoding of mixed nuclei populations.

Forward and reverse genetics have long advanced plant molecular biology. While several plant mutants have been used in single‐cell omics, the full potential of genetics in single‐cell and spatial omics remains largely untapped (Birnbaum, 2018). Recent advances, primarily in animal systems, now enable high‐content genetic screening using single‐cell and spatial omics readout. CRISPR‐based functional genomics has been integrated with single‐cell omics to dissect gene function and regulatory networks at unprecedented scale (Fig. 5a). Perturb‐seq and Perturb‐ATAC combine CRISPR‐based genetic perturbations with scRNA‐seq and scATAC‐seq, respectively, to study gene function and regulatory networks in cell cultures or *in vivo* (Dixit *et al*., 2016; Rubin *et al*., 2019; Jin *et al*., 2020). PerturbMap introduces dozens of CRISPR perturbations and characterises phenotypes using imaging and spatial transcriptomics (Dhainaut *et al*., 2022). Perturb‐multi integrates single‐cell transcriptomics and spatial omics with *in vivo* CRISPR perturbations for comprehensive gene function analysis (Saunders *et al*., 2024). Experimental and computational innovations are beginning to answer fundamental biological questions: how to link genes, cells, and tissues, and the mechanisms underpinning these linkages (Rood *et al*., 2024).

Chemical genetics offer unbiased ways to investigate the function of gene products by perturbing specific functions of proteins or metabolites using small molecules. For instance, Herrmann *et al*. (2025) identified a small molecule, kC9, that inhibits MPK6 function, revealing unexpected signalling crosstalk between development and immune responses in plant guard cells. The sci‐Plex platform demonstrated the power of chemical genetics for high‐throughput single‐cell screening. Using combinatorial indexing‐based snRNA‐seq combined with nuclei hashing, Srivatsan *et al*. (2020) analysed transcriptome responses of three cancer cell lines exposed to 188 compounds, which enables the screening of thousands of conditions in a single experiment (Fig. 5b). sci‐Plex‐GxE combines sci‐Plex with CRISPR perturbations, enabling simultaneous single‐cell genetic and chemical screening at scale. This method tested the contribution of 522 human kinases to drug response (McFaline‐Figueroa *et al*., 2024). Such high‐throughput, high‐content (global transcription), and high‐resolution (single‐cell) molecular phenotyping approaches hold significant potential for revealing cell‐type/state‐specific contributions of genes and signalling pathways in plants.

## IV. Challenges and opportunities for plant research in adopting new single‐cell and spatial omics technologies

While challenges in plant sc/snRNA‐seq are well‐documented (Seyfferth *et al*., 2021; Grones *et al*., 2024), emerging methods still lack established community guidelines. Critical areas requiring development include experimental design (e.g. biological replicates), sample treatment, preprocessing, data acquisition, analysis, and sharing.

Mammalian research benefits from scalable single‐cell analyses, such as perturbation screening, using cell culture systems. However, such applications remain scarce in plant research. Single‐cell and spatial perturbation assays in mammalian systems often take advantage of adeno‐associated viruses to deliver guide RNAs for CRISPR‐based gene editing. By contrast, efficient viral delivery methods for plants remain underdeveloped, posing a bottleneck. Developing robust plant cell culture models and molecular delivery systems suitable for high‐throughput single‐cell screening will drive the discoveries of cell‐specific gene regulatory mechanisms. For instance, nanoparticles offer an alternative vehicle for molecular delivery into plants (Demirer *et al*., 2021).

The presence of the cell wall complicates sample preparation, such as protoplasting for single‐cell omics and tissue permeabilisation for spatial omics of thick tissue samples. Single‐nucleus approaches can bypass the need for protoplasting, and tissue sections can in some cases substitute for whole‐mount imaging in spatial omics. Ming *et al*. (2025) recently developed FX‐Cell, a scRNA‐seq method involving tissue fixation before protoplasting, improving protoplast yield from difficult tissues while minimising transcriptomic changes. This approach offers a compelling alternative that reshapes the discussion on protoplast‐ vs nuclei‐based strategies in plant single‐cell transcriptomics (Grones *et al*., 2024). Single‐cell proteomics, metabolomics, and ExM require intact protoplasts or permeabilised tissues. Developing effective cell wall degradation strategies is critical, but existing protocols are limited to a few model tissues. Given the diversity of cell wall composition across plant species and tissues, these protocols must be extended to cover plant diversity. Plant‐associated microbes, which produce a variety of cell wall‐degrading enzymes, offer a potential resource for identifying or engineering new enzymes. Experimental evolution strategies could expand the repertoire of these enzymes, facilitating broader applications of single‐cell and spatial omics in plants.

The plant science community has access to extensive genetic resources, including natural accessions, inbred lines, and mutants. Single‐cell and spatial omics approaches that do not require transgenic materials offer a direct way to leverage these rich genetic resources. Cost‐effective sample multiplexing strategies will be key to analysing diverse genotypes efficiently. High‐resolution molecular analyses across diverse genotypes can reveal the evolution and adaptation of cell types and states.

While RNA‐ and DNA‐based single‐cell omics are largely compatible with plant tissues, protein‐based approaches remain limited by the lack of high‐quality, validated antibodies for many proteins in plants. Advances in AI‐driven antibody design, inspired by progress in therapeutic antibody development (Kim *et al*., 2023), offer potential solutions. Alternative strategies include aptamer‐ and nanobody‐based detection. Expanding the repertoire of plant‐compatible antibodies could unlock various applications of single‐cell and spatial (multi)omics approaches in plants.

Parallel advancements in computational tools must accompany experimental innovations. Community guidelines and benchmarking practices are vital for robustness and reproducibility in single‐cell and spatial omics. Recent efforts in the animal field have produced comprehensive best‐practice workflows and benchmarking frameworks in single‐cell and spatial omics (Heumos *et al*., 2023). These resources provide valuable guidance on tool selection, preprocessing, integration, and interpretation strategies. However, such community‐driven standardisation remains largely absent in the plant field, in which the diversity of genome structures and tissue architectures poses unique challenges. Adapting and expanding these frameworks to plant‐specific contexts will be essential for advancing computational methodology and facilitating cross‐study comparisons.

Effective data sharing is also crucial as studies generate increasingly large datasets that exceed their immediate scope of analysis. In addition to providing raw data and analysis code with proper metadata, building reference atlases is vital for maximising the utility of these datasets. Key steps include the following: (1) selecting focus areas and datasets; (2) data preprocessing; (3) metadata harmonisation and quality control; (4) data integration; (5) atlas evaluation and reannotation; and (6) sharing and extending the atlas (Hrovatin *et al*., 2024). Continuous and transparent community discussions on these steps are essential to ensuring that the cell atlases keep evolving and catalyse new discoveries in plant research.

## V. Conclusions and outlook

This review highlighted how emerging single‐cell and spatial omics technologies advance our understanding of plant cellular complexity in spatiotemporal contexts and enable cell‐specific functional analyses. These advances will transform our understanding of plant cell types, states, and their dynamic interactions underlying plant behaviour.

How far can we push the boundaries of cellular measurements? How will this ultimately help us understand plant biology? The excitement surrounding the continuous development of new technologies is undeniable, but real biological discoveries are made when biological questions guide the selection of methods, the design of experiments, and the refinement of technologies. The increasingly multidisciplinary nature of plant biology demands robust community support for knowledge and resource exchange, including reference atlases, detailed protocols, bioinformatics pipelines, identified knowledge gaps, and shared instrumentation. Communities such as the Plant Cell Atlas play crucial roles in catalysing biology–technology interactions (Plant Cell Atlas Consortium *et al*., 2021).

The field of plant biology stands at an exciting juncture, in which emerging single‐cell and spatial omics technologies are opening new avenues to tackle long‐standing questions and enabling the formulation of entirely new ones. Synergy between innovative technologies and targeted biological questions promises to propel our understanding of plant biology in the coming years.

## Competing interests

None declared.

## Disclaimer

The New Phytologist Foundation remains neutral with regard to jurisdictional claims in maps and in any institutional affiliations.
